# Simplified intravoxel incoherent motion DWI for differentiating malignant from benign breast lesions

**DOI:** 10.1186/s41747-022-00298-6

**Published:** 2022-09-29

**Authors:** Petra Mürtz, Mark Tsesarskiy, Alois M. Sprinkart, Wolfgang Block, Oleksandr Savchenko, Julian A. Luetkens, Ulrike Attenberger, Claus C. Pieper

**Affiliations:** 1 Department of Diagnostic and Interventional Radiology, University Hospital Bonn, Venusberg-Campus 1, 53127 Bonn, Germany; 2 Department of Radiotherapy and Radiation Oncology, University Hospital Bonn, Venusberg-Campus 1, 53127 Bonn, Germany; 3 Department of Neuroradiology, University Hospital Bonn, Venusberg-Campus 1, 53127 Bonn, Germany

**Keywords:** Breast neoplasms, Diffusion magnetic resonance imaging, Feasibility studies, Perfusion, Sensitivity and specificity

## Abstract

**Background:**

To evaluate simplified intravoxel incoherent motion (IVIM) diffusion-weighted imaging (DWI) for differentiating malignant *versus* benign breast lesions as (i) stand-alone tool and (ii) add-on to dynamic contrast-enhanced magnetic resonance imaging.

**Methods:**

1.5-T DWI data (*b* = 0, 50, 250, 800 s/mm^2^) were retrospectively analysed for 126 patients with malignant or benign breast lesions. Apparent diffusion coefficient (ADC) ADC (0, 800) and IVIM-based parameters *D*_1_′ = ADC (50, 800), *D*_2_′ = ADC (250, 800), *f*_1_′ = *f* (0, 50, 800), *f*_2_′ = *f* (0, 250, 800) and D*′ = D* (0, 50, 250, 800) were voxel-wise calculated without fitting procedures. Regions of interest were analysed in vital tumour and perfusion hot spots. Beside the single parameters, the combined use of *D*_1_′ with *f*_1_′ and *D*_2_′ with *f*_2_′ was evaluated. Lesion differentiation was investigated for lesions (i) with hyperintensity on DWI with *b* = 800 s/mm^2^ (*n* = 191) and (ii) with suspicious contrast-enhancement (*n* = 135).

**Results:**

All lesions with suspicious contrast-enhancement appeared also hyperintense on DWI with *b* = 800 s/mm^2^. For task (i), best discrimination was reached for the combination of *D*_1_′ and *f*_1_′ using perfusion hot spot regions-of-interest (accuracy 93.7%), which was higher than that of ADC (86.9%, *p* = 0.003) and single IVIM parameters *D*_1_′ (88.0%) and *f*_1_′ (87.4%). For task (ii), best discrimination was reached for single parameter *D*_1_′ using perfusion hot spot regions-of-interest (92.6%), which were slightly but not significantly better than that of ADC (91.1%) and *D*_2_′ (88.1%). Adding *f*_1_′ to *D*_1_′ did not improve discrimination.

**Conclusions:**

IVIM analysis yielded a higher accuracy than ADC. If stand-alone DWI is used, perfusion analysis is of special relevance.

## Key points

- Various placement and analysis methods were evaluated for simplified intravoxel incoherent motion (IVIM) diffusion-weighted magnetic resonance imaging of breast lesions.
- High diagnostic accuracy (93.7%) was achieved for malignant *versus* benign breast lesion assessment, higher than that of apparent diffusion coefficient (86.9%).
- Simplified IVIM is suitable for clinical application in breast imaging.

## Background

Dynamic contrast-enhanced magnetic resonance imaging (DCE-MRI) of the breast is known for its high sensitivity in the detection of breast cancer. Specificity, however, is typically below or around 80% implying the possibility of unnecessary biopsies [1]. Diffusion-weighted imaging (DWI) with analysis of apparent diffusion coefficient (ADC) is increasingly being incorporated into routine breast protocols as an adjunct to DCE-MRI in order to improve diagnostic specificity [2–8]. Furthermore, DWI may potentially substitute delayed phase DCE-MRI [9], allow for early assessment and prediction of treatment effects [4, 6] and may have potential as a stand-alone screening tool for breast cancer [6, 10].

The analysis of DWI by ADC determination does not take into account that the diffusion-weighted signal is not only influenced by tissue water diffusion, but also by blood flow within the capillary network. By the intravoxel incoherent motion (IVIM) concept, the diffusion-weighted signal is described biexponentially as weighted sum (perfusion fraction *f*) of signal components influenced by motion of water molecules in tissue (‘true’ diffusion coefficient *D*) and in the blood vessels (pseudodiffusion coefficient D*) depending on the diffusion-weighting factor (*b* value) [11]. D* depends on blood flow velocity and capillary segment length [11]. IVIM is of special interest in oncology [12], especially in situations where diffusion and perfusion oppositely influence the DWI signal decay and also the ADC. This happens, for example, in distinguishing malignant from benign breast lesions: a decreased *D* together with an increased *f* may lead to underestimation of diffusion reduction by ADC [13–24]. The same is for monitoring response to neoadjuvant therapy: an increased *D* together with decreased *f* may lead to underestimation of diffusion increase by ADC [25–27].

Conversely, in the IVIM approach, normally fully unconstrained nonlinear least squares fitting procedures are used for simultaneous determination of *D*, *f* and D*. The use of fitting procedures requires the acquisition of DWI sequences with many different *b* values and sufficient signal-to-noise ratios, resulting into long acquisition times. In addition, they often lead to numerical instabilities, poor reproducibility and unreliable parameter values of *f* and D* in the tissue with low perfusion [28] such as normal fibroglandular tissue and cysts [22, 23, 29]. Improved stability can be achieved by segmented fitting [5, 16, 22, 24, 25, 30–32] or simplified IVIM [3, 33–40]. Both approaches are based on the assumption that the pseudodiffusion component has essentially decayed to zero for *b* values above a suitably high threshold. However, with simplified IVIM, parameters are determined by explicit formulas and not by fitting. Moreover, for simplified IVIM, the acquisition of DWI sequences with only three or four different *b* values is needed. To our knowledge, the application of simplified IVIM for breast lesion differentiation was only evaluated in one initial study based on three *b* values and including only a small cohort of 26 patients [3].

Thus, the aim of this study was to evaluate simplified IVIM for malignant *versus* benign differentiation of breast lesions on a larger patient cohort. Furthermore, by using four *b* values, different IVIM parameter estimates and combinations could be investigated. DWI was tested (i) as a stand-alone tool using all lesions with hyperintensity on b800 DWI and (ii) as an add-on to DCE-MRI for all lesions with suspicious contrast enhancement.

## Methods

### Subjects

This retrospective study was approved by the local institutional review board of the University Hospital Bonn, which waived the need for informed patient consent. Over a period of 34 months (from August 2012 to November 2014 and from October 2017 to March 2018), 180 consecutive patients were examined with a standardised breast imaging protocol and with DWI. According to the recommendations of the European Society of Breast Cancer Specialists working group (EUSOMA) [41] and to the national German guidelines [https://www.awmf.org/leitlinien/detail/ll/032-045OL.html], MRI was performed to increase the diagnostic accuracy in cancer detection, which concerns patients with unclear mammography/ultrasound findings (34%), follow-up of former cancer patients (1%) and preventive screening of high-risk women (< 1%), or to get a staging before treatment decision (34%), before surgery (20%), or before preoperative treatment (10%).

Of those 180 patients, 134 (74.4%) were elected fulfilling the inclusion criteria of having one or more lesions suspected on DWI with *b* = 800 s/mm^2^ or DCE-MRI (see below), which all had a confirmed diagnosis, and of having not yet received a neoadjuvant therapy or radiation treatment. Of these, patients were excluded if they had implants (*n* = 3), if the lesions were less than 8 mm in size to a avoid partial volume effects [2] (*n* = 4), or if the quality of DWI was insufficient due to pixel misalignments (*n* = 1). None of the patients was excluded because of lesions were visible only on DCE-MRI and not on DWI. Data regarding the remaining 126 female patients (age 54 ± 12 years, mean ± standard deviation; range 25−82 years) were analysed, 95 patients with malignant lesions and 31 patients exclusively with benign lesions that were only suspected at DWI with *b* = 800 s/mm^2^. The diagnosis of lesions with suspicious contrast enhancement according to the morphologic and kinetic features defined in the Breast Imaging-Reporting and Data System (BI-RADS) MRI lexicon [42] was established on the basis of histopathological examination according to the World Health Organization classification of breast tumours [43] or follow-up investigations with a minimum interval time of 12 months. Benignancy in lesions visible on DWI and with no or nonsuspicious contrast enhancement was established by DCE-MRI and confirmed by ultrasound and follow-up with the exception of 4 patients, where no follow-up was available. Details are given in Table 1.

**Table 1:** Overview of lesion types (*n* = 191)

Group	Type of lesion	Number
A	Benign lesions with no or non-suspicious contrast-enhancement:	56
	1) Simple cyst (*n* = 20), seroma after biopsy or surgery (*n* = 3)	23
	2) Complicated (haemorrhagic/proteinaceous) cyst	6
	3) Haematoma	7
	4) Normal fibroglandular tissue without contrast-enhancement	14
	5) Normal fibroglandular tissue with non-suspicious contrast-enhancement	6
B	Benign lesions with suspicious contrast-enhancement:	30
	6) Fibroadenoma (*n* = 11), fibrocystic mastopathy (*n* = 10), adenomyoepithelioma (*n* = 1)	22
	7) Syringomatous adenoma (n = 1), intraductal papilloma (*n* = 1), sclerosing adenosis (*n =* 1)	3
	8) Flat epithelial atypia	1
	9) Intramammary lymph node	4
C	Malignant lesions with suspicious contrast-enhancement:	105
	10) Invasive carcinoma G1 (6 ductal, 1 tubular)	7
	11) Invasive carcinoma G2 (23 ductal, 16 lobular, 1 ductolobular, 1 ductal mucinous, 1 other)	42
	12) Invasive carcinoma G3 (34 ductal, 1 lobular, 2 mixed, 2 necrotic, 4 other)	43
	13) Invasive carcinoma with unknown grading (1 ductal)	1
	14) Ductal carcinoma *in situ* (1 G2, 9 G3)	10
	15) Intramammary lymph node metastases	2

Group A was composed of benign lesions with hyperintensity on diffusion-weighted imaging (DWI) with *b* = 800 s/mm^2^ and no or non-suspicious contrast-enhancement according to the morphologic and kinetic features defined in the Breast Imaging-Reporting and Data System MRI lexicon [42] (*n =* 56). Group B was composed of benign lesions with suspicious contrast-enhancement (*n =* 30). Group C was composed of malignant lesions, which were all with suspicious contrast-enhancement (*n =* 105). All lesions with suspicious contrast enhancement (groups B and C) were hyperintense on DWI with *b* = 800 s/mm^2^. The diagnosis of group B and C lesions was established on the basis of histopathological examination of surgical or core needle biopsy according to the World Health Organization classification of breast tumours [43] or follow-up investigations with a minimum interval time of 12 months. The diagnosis of group A lesions was established by DCE-MRI and confirmed by ultrasound and follow-up with the exception of 4 patients (no follow-up avilable)

*DCE* Dynamic contrast enhancement, *MRI* Magnetic resonance imaging

### Magnetic resonance imaging protocol

A single-shot spin-echo echo-planar DWI variant (Table 2) was acquired before contrast agent injection on a clinical 1.5-T MRI scanner (ACS-NT, 1.5 T; Philips Healthcare, Best, Netherlands; gradient system: maximum amplitude of 30 mT/m, maximum slew rate of 150 T/m/s) using a commercially available four-element phased-array surface receiver coil for signal detection. Isotropic diffusion-weighted images were reconstructed directly on the MRI system.

**Table 2:** Technical parameters of the diffusion-weighted imaging sequence

Name	Value
FOV (RL × AP)/orientation	400 × 300 mm/transversal
Slice number/thickness/gap	29/4.0 mm/−1.0 mm
Matrix/pixel size	132 × 101/3.0 × 3.0 mm
Echo time	60 ms
Repetition time	2116 ms
EPI factor/half-Fourier factor/SENSE factor	55/0.6/2
Diffusion gradients	Three orthogonal directions
Duration/distance	22.6/31.9 ms
*b* values (number of excitations)	0, 50, 250 s/mm^2^ (3), 800 s/mm^2^ (6)
Fat suppression method	STIR (inversion time = 180 ms)
Water-fat shift/bandwidth	7.1 pixel/30.4 Hz
Bandwidth in EPI frequency direction	2203.5 Hz
Acquisition time	2:53 min:s

*AP* Anterior-posterior, *EPI* Echo-planar imaging, *FOV* Field of view, *RL* Right-left, *SENSE* Parallel imaging with sensitivity encoding, *STIR* Short-time inversion recovery

DCE imaging (31 slices, field of view 290–380 × 320–420 mm, spatial resolution of 0.9–2.1 × 0.9–2.1× 3 mm, repetition time/echo time 262/4.4 ms, flip angle 90°) was performed prior to and four times after a bolus injection of gadobutrol (Gadovist, Bayer, Leverkusen, Germany) at 0.1 mmol/kg body weight, followed by a saline flush, all injected at 3 mL/s. Subtraction and maximum intensity projection images were then obtained.

### Postprocessing

According to IVIM theory [11], a two-compartment model of extravascular and intravascular space and a biexponential approach of the signal attenuation was assumed:

1 $$S(b)/S(0)=f\cdot \mathit{\exp}\left(\hbox{-} b\cdot {D}^{\ast}\right)+\left( 1\hbox{-} f\right)\cdot \mathit{\exp}\left(\hbox{-} b\cdot D\right)$$

For analysis [33, 34], two different approximations of *D* and *f* were calculated from signal intensities S(b) and S(0), one using *b*_0_=0, *b*_1_=50, *b*_3_=800 and one using *b*_0_=0, *b*_2_=250, *b*_3_=800 s/mm^2^:

2 $${D}_1^{\hbox{'}}= ADC\left(50,800\right)=\frac{\ln \left(S\left({b}_1\right)\right)-\ln \left(S\left({b}_3\right)\right)}{b_3-{b}_1}$$

3 $${D}_2^{\hbox{'}}= ADC\left(250,800\right)=\frac{\ln \left(S\left({b}_2\right)\right)-\ln \left(S\left({b}_3\right)\right)}{b_3-{b}_2}$$

4 $${f}_1^{\hbox{'}}=f\left(0,50,800\right)=1-\frac{S\left({b}_1\right)}{S(0)}\cdot {\exp}^{D_1^{\hbox{'}}\cdot {b}_1}$$

5 $${f}_2^{\hbox{'}}=f\left(0,250,800\right)=1-\frac{S\left({b}_2\right)}{S(0)}\cdot {\exp}^{D_2^{\hbox{'}}\cdot {b}_2}$$

From four *b* values, D* was approximated by using *D*_2_′ and *f*_2_′ and the reading for *b*_1_:

6 $${D}^{\ast \hbox{'}}={D}^{\ast}\left(0,50,250,800\right)=-\frac{1}{b_1}\cdot \ln \left[\frac{1}{f_2^{\hbox{'}}}\cdot \left(\frac{S\left({b}_1\right)}{S(0)}-\left(1-{f}_2^{\hbox{'}}\right)\cdot \mathrm{ex}{\mathrm{p}}^{-{D}_2^{\hbox{'}}\cdot {b}_1}\right)\right]$$

The conventional ADC was calculated:

7 $$ADC= ADC\left(0,800\right)=\frac{\ln \left(S\left({b}_0\right)\right)-\ln \left(S\left({b}_3\right)\right)}{b_3-{b}_0}$$

Parameter maps were calculated offline in MATLAB (MathWorks, Natick, MA, USA).

### Image analysis

Image analysis was performed by a radiologist (C.C.P.) with more than 10 years of experience in breast imaging and a physicist (P.M.) with more than 20 years of experience in DWI. The regions of interests (ROIs) were placed in consensus by the two readers. In each patient included, all lesions with dimensions larger than 8 mm, visible on at least three slices of DWI, were analysed. In the final analysis, only the largest lesion of each type per patient (Table 1) was included. For each lesion, a region of interest (ROI) was placed on a central slice of DWI image obtained with *b* = 800 s/mm^2^ that was largely unaffected by residual fat signal, susceptibility artefacts and pixel misalignments. The hand-drawn ROI was adapted to the hyperintense structures of the lesion, referred to as ‘vital tumour’ ROI (VT-ROI). Areas close to the rim and centrally deviating areas in DWI, which may be necrotic or haemorrhagic parts, cystic components and mucous, were excluded. After the anatomical position was visually cross-checked for pixel misalignments between images with different *b* values, the ROI was copied into the parameter maps. Compared to ROIs in areas of diffusion restriction, analysis of ROIs in perfusion hot spots may improve diagnostic accuracy (see the ‘Discussion’ section). Thus, a second ROI was placed on the perfusion fraction maps within an area of high perfusion (and low diffusion if possible), referred to as ‘hot spot’ ROI (HS-ROI). If no hot spot could be identified (homogeneous lesions), the VT-ROI was re-used.

### Statistical analysis

According to the normal or non-normal distribution, continuous data are given as mean ± standard deviation or median and interquartile range (IQR, 25−75th percentile). Due to non-normal distribution, differences between lesion groups were tested using the Mann-Whitney *U* test (SPSS, version 24.0, IBM, Armonk, NY, USA) with a statistical significance set as < 0.05. Receiver operating characteristic (ROC) analysis (pROC package in *R*, version 1.17.0.1, GNU project, Boston, MA, USA [44]) was performed to evaluate the discrimination ability of the parameters (ADC, *D*_1_′, *D*_2_′, *f*_1_′, *f*_2_′, D*′). The DeLong method was used to compare the area under the curve (AUC) of dependent ROC curves [45]. Optimal cutoff value of each parameter was calculated for maximum Youden’s index. Sensitivity, specificity and accuracy (rate of correctly identified cases) with their 95% confidence interval (CI) were calculated for each parameter and for the combined use of *D*_1_′ and *f*_1_′ (*D*_1_′+*f*_1_′) and *D*_2_′ and *f*_2_′ (*D*_2_′+*f*_2_′). Hereby, lesions were assigned as malignant, if both parameters (*D*_i_′ and *f*_i_′, *i* = 1, 2) fulfilled the criterion of malignancy based on the cutoff values determined for the single parameters; otherwise, they were assigned as benign.

## Results

**Fig. 1:**
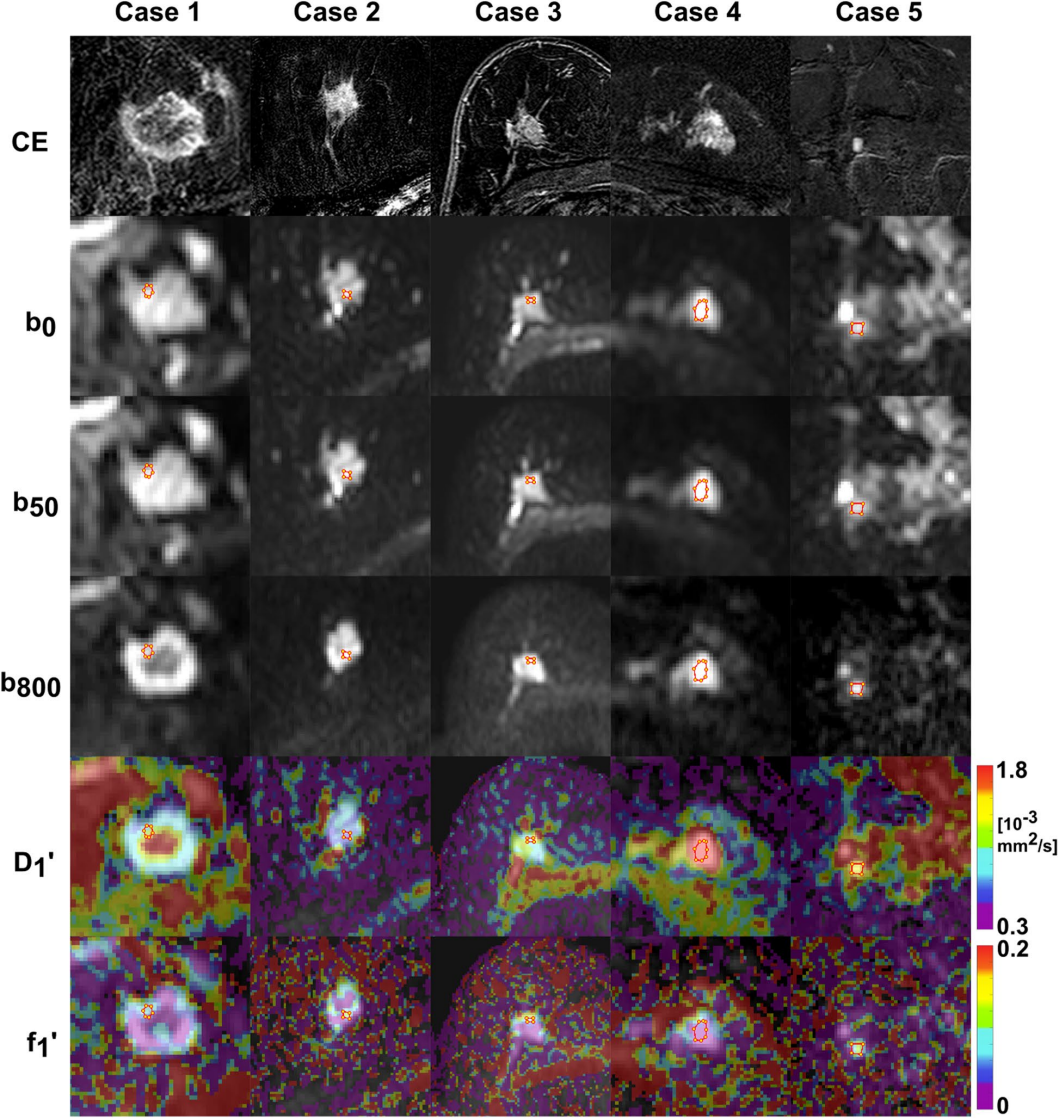
Examples of axial diffusion-weighted imaging and intravoxel incoherent motion-parameter maps of breast lesions. From top to bottom, contrast-enhanced T1-weighted subtraction images (CE), original trace-weighted diffusion-weighted (DW) images with *b* = 0, 50, 800 s/mm^2^, and *D*_1_′ and *f*_1_′ colour-coded maps overlaid to DW images with *b* = 800 s/mm^2^ are given together with the used hot spot regions-of-interest (HS-ROIs). Invasive ductal carcinoma (case 1, 2 and 3, all G3 grade) typically show *D*_1_′ values between 0.6 and 1.2 × 10^-3^ mm^2^/s in areas with maximum hyperintensity on DW images with *b* = 800 s/mm^2^ (turquoise) and mixed *f*_1_′ values with hot spot values between 0.05 and 0.15 (turquoise). In case 1, central necrosis is present showing high *D*_1_′ values (red-yellow). Hyperintense area in case 4 was histologically diagnosed as fibrous mastopathy, ductal and lobular hyperplasia showing high *D*_1_′ of 2.0 × 10^-3^ mm^2^/s (red) and low *f*_1_′ of 0.01 (turquoise). Another case with fibrous mastopathy (case 5) showed also high *D*_1_′ of 1.4 × 10^-3^ mm^2^/s (yellow) but higher *f*_1_′ of 0.08

**Fig. 2:**
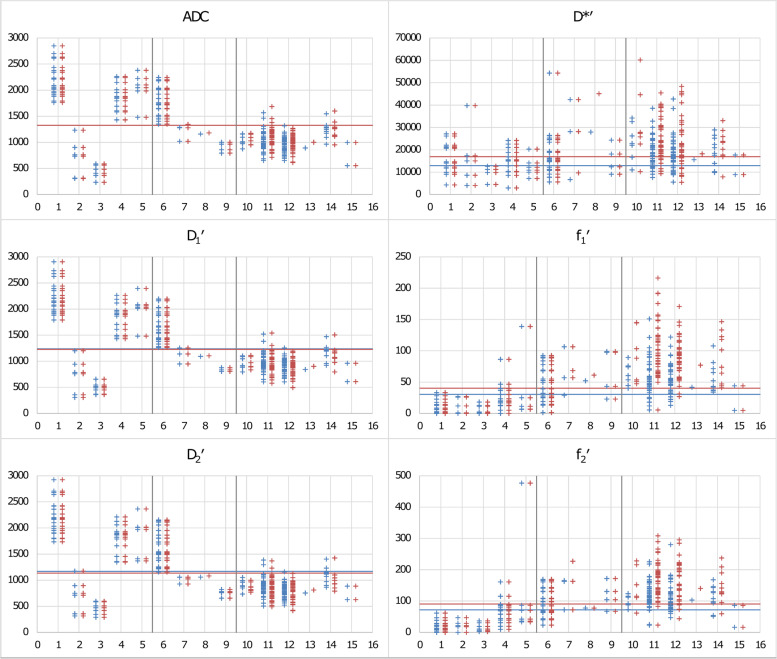
Overview to ADC, *D*_1_′, *D*_2_′, *f*_1_′, *f*_2_′, and D*′ values grouped according to different lesion types. The group and subgroup designations are explained in Table 1. Blue data points belong to vital tumour regions of interest (VT-ROIs), red to hot spot ROIs (HS-ROIs). For each parameter, the cutoff points (see Tables 5 and 6) are also given, as blue line for VT-ROIs and red line for HS-ROIs. Apparent diffusion coefficient (ADC), *D*_1_′, *D*_2_′, and D*′ are given in units of 10^-6^ mm^2^/s, *f*_1_′, and *f*_2_′ values are given in units of 10^-3^. It can be seen that values of ADC, *D*_1_′, and *D*_2_′ were typically smaller for group C compared to group A+B, for VT- and HS-ROIs. Exceptions were complicated (haemorrhagic/proteinaceous) cyst and haematoma, which also had low values. Values of *f*_1_′ and *f*_2_′ were typically larger for group C compared to group A+B, especially for HS-ROIs. However, many benign lesions with suspicious contrast enhancement (group B) also have large values

In the 126 female patients, a total of 191 lesions were analysed (Table 1). Of 135 lesions that showed suspicious contrast-enhancement, 30 were benign (group B) and 105 malignant (group C). All lesions with suspicious contrast-enhancement appeared hyperintense on DWI with *b* = 800 s/mm^2^. In particular, this means that for malignant lesions, a detection rate of 100% was reached. Further 56 lesions were hyperintense on DWI with *b* = 800 s/mm^2^ but showed no or non-suspicious contrast-enhancement and were all benign (group A). The VT-ROI size ranged from 4 to 673 mm^2^ (median 35, IQR 19−81) while the HS-ROI size ranged from 2 to 349 mm^2^ (median 12, IQR 6−24). The median percentage of perfused voxels (*i.e.*, voxels with a defined D*′) in the VT-ROIs was 25 (IQR 4−50) for group A, 68 (IQR 50−90) for group B and 75 (IQR 57−89) for group C (*p* < 0.001 for A *versus* B and A *versus* C; the difference was not significant for B *versus* C (*p* = 0.372). Maximum perfusion was not always in areas of minimum diffusion. Example images are shown in Fig. 1. An overview of all measured parameter values is given in Fig. 2. Mean parameter values of group A, B, A+B, and C are given in Tables 3 and 4. Lesions of groups A, B and C were analysed for the evaluation of IVIM as a stand-alone tool, lesions of groups B and C for the evaluation of IVIM as an add-on to DCE-MRI.

**Table 3:** Apparent diffusion coefficient (ADC) and intravoxel incoherent motion parameter values for benign lesions with hyperintensity on DWI with *b* = 800 s/mm^2^ and no or non-suspicious contrast-enhancement (group A, *n =* 56), benign lesions with suspicious contrast-enhancement (group B, *n =* 30) and malignant lesions, which were all with suspicious contrast-enhancement (group C, *n =* 105)

Group	ADC	*D*_1_'	*D*_2_'	*f*_1_'	*f*_2_'	D*'
Vital tumour ROIs						
A	1,705 ± 704 (*n =* 56)	1,721 ± 699 (*n =* 56)	1,680 ± 707 (*n =* 56)	16 ± 23 (*n =* 56)	41 ± 68 (*n =* 56)	14,960 ± 7,279 (*n =* 43)
B	1,575 ± 421 (*n =* 30)	1,519 ± 442 (*n =* 30)	1,435 ± 437 (*n =* 30)	50 ± 31 (*n =* 30)	106 ± 44 (*n =* 30)	19,130 ± 10,634 (*n =* 30)
A+B	1,660 ± 621 (*n =* 86)	1,650 ± 626 (*n =* 86)	1,595 ± 634 (*n =* 86)	28 ± 31 (*n =* 86)	64 ± 68 (*n =* 86)	16,674 ± 8,985 (*n =* 73)
C	1,021 ± 181 (*n =* 105)	957 ± 173 (*n =* 105)	871 ± 172 (*n =* 105)	56 ± 26 (*n =* 105)	113 ± 42 (*n =* 105)	19,046 ± 6,997 (*n =* 105)
A+B+C	1,308 ± 540 (*n =* 191)	1,269 ± 558 (*n =* 191)	1,197 ± 571 (*n =* 191)	43 ± 32 (*n =* 191)	91 ± 60 (*n =* 191)	18,073 ± 7,936 (*n =* 178)
Hot spot ROIs						
A	1,705 ± 704 (*n =* 56)	1,721 ± 699 (*n =* 56)	1,680 ± 707 (*n =* 56)	16 ± 23 (*n =* 56)	41 ± 68 (*n =* 56)	14,960 ± 7,279 (*n =* 43)
B	1,578 ± 419 (*n =* 30)	1,519 ± 442 (*n =* 30)	1,435 ± 437 (*n =* 30)	52 ± 31 (*n =* 30)	108 ± 48 (*n =* 30)	19,798 ± 11,442 (*n =* 30)
A+B	1,661 ± 620 (*n =* 86)	1,650 ± 626 (*n =* 86)	1,595 ± 635 (*n =* 86)	28 ± 31 (*n =* 86)	65 ± 69 (*n =* 86)	16,948 ± 9,454 (*n =* 73)
C	1,057 ± 192 (*n =* 105)	933 ± 187 (*n =* 105)	835 ± 189 (*n =* 105)	94 ± 39 (*n =* 105)	161 ± 59 (*n =* 105)	23,762 ± 10,431 (*n =* 105)
A+B+C	1,329 ± 532 (*n =* 191)	1,256 ± 568 (*n =* 191)	1,177 ± 586 (*n =* 191)	65 ± 48 (*n =* 191)	118 ± 80 (*n =* 191)	20,968 ± 10,563 (*n =* 178)

Mean values ± standard deviations for vital tumour regions of interest (ROIs) and hot spot ROIs. ADC, *D*_1_′, *D*_2_′, and D*′ are given in units of 10^-6^ mm^2^/s, *f*_1_′ and *f*_2_′, values are given in units of 10^-3^

**Table 4:** Statistical comparisons (*p* values) for the results shown in Table 3

Comparison	ADC	*D*_1_'	*D*_2_'	*f*_1_'	*f*_2_'	D*'
Vital tumour ROIs						
A *versus* B	0.062	0.036	0.022	*<* 0.001	*<* 0.001	0.078
A *versus* C	*< *0.001	*<* 0.001	*<* 0.001	*<* 0.001	*<* 0.001	0.001
B *versus* C	*< *0.001	*<* 0.001	*<* 0.001	0.305	0.446	0.641
A+B *versus* C	< 0.001	*<* 0.001	*<* 0.001	*<* 0.001	*<* 0.001	0.010
Hot spot ROIs						
A *versus* B	0.062	0.036	0.022	*<* 0.001	*<* 0.001	0.069
A *versus* C	*< *0.001	*<* 0.001	*<* 0.001	*<* 0.001	*<* 0.001	*<* 0.001
B *versus* C	< 0.001	*<* 0.001	*<* 0.001	*<* 0.001	*<* 0.001	0.027
A+B *versus* C	< 0.001	*<* 0.001	*<* 0.001	*<* 0.001	*<* 0.001	*<* 0.001

See Table 3 for abbreviations

### IVIM for stand-alone differentiation of malignant from benign and (group C *versus* group A+B)

Values of ADC, *D*_1_′, and *D*_2_′ were significantly smaller and values of *f*_1_′, *f*_2_′, and D*′ were significantly larger for group C compared to A+B, for VT- and HS-ROIs (Table 5).

**Table 5:** Results of the receiver operating characteristic analysis for the differentiation between benign and malignant lesions for group A+B *versus* group C

Test variable	Numbers	AUC	Standard error^a^	Asym. sign.	Asym. 95% CI		Cutoff point	Sensitivity	Specificity	Accuracy
					Lower bound	Upper bound				
Vital tumour ROIs										
ADC	86 *versus* 105	0.817	0.039	< 0.001	0.740	0.893	1,328.5	0.971	0.756	0.874
*D*_1_′	86 *versus* 105	0.819	0.038	< 0.001	0.743	0.894	1,238.7	0.952	0.767	0.869
*D*_2_′	86 *versus* 105	0.826	0.037	< 0.001	0.752	0.900	1,167.8	0.962	0.756	0.869
*f*_1_′ #	86 *versus* 105	0.795	0.035	< 0.001	0.726	0.865	30.6	0.876	0.709	0.801
*f*_2_′ #	86 *versus* 105	0.789	0.036	< 0.001	0.719	0.860	72.2	0.895	0.674	0.796
D*′ #	73 *versus* 105	0.613	0.044	0.010	0.526	0.700	12,912.4	0.810	0.425	0.652
*D*_1_′+f_1_′	86 *versus* 105						1,238.7/30.6	0.829	0.930	0.874
*D*_2_′+*f*_2_′	86 *versus* 105						1,167.8/72.2	0.867	0.919	0.890
Hot spot ROIs										
ADC	86 *versus* 105	0.812	0.039	< 0.001	0.734	0.889	1,327.2	0.952	0.767	0.869
*D*_1_′	86 *versus* 105	0.826	0.037	< 0.001	0.753	0.900	1,229.2	0.971	0.767	0.880
*D*_2_′	86 *versus* 105	0.836	0.036	< 0.001	0.765	0.907	1,135.1	0.971	0.767	0.880
*f*_1_′ #	86 *versus *105	0.905	0.023	< 0.001	0.860	0.950	40.5	0.971	0.756	0.874
*f*_2_′ #	86 *versus* 105	0.881	0.026	< 0.001	0.830	0.932	90.7	0.905	0.767	0.843
D*′ #	73 *versus* 105	0.707	0.040	< 0.001	0.629	0.785	16,900.6	0.771	0.603	0.702
*D*_1_′+*f*_1_′	86 *versus* 105						1,229.2/40.5	0.943	0.930	0.937
*D*_2_′+*f*_2_′	86 *versus* 105						1,135.1/90.7	0.876	0.942	0.906

The optimal cutoff point according the Youden index is given in 10^-6^ mm^2^/s for ADC, *D*_1_′, *D*_2_′, and D*′ and in 10^-3^ for *f*_1_′ and *f*_2_′, whereby a lower test result indicates a more ‘positive’ test (a negative test direction is marked with #). Sensitivity, specificity and accuracy are given for each parameter and for the combined parameter use *D*_1_′+*f*_1_′ and *D*_2_′+*f*_2_′, whereby lesions were assigned as malignant, if both parameters fulfilled the criterion of malignancy using the cutoff values determined for the single parameters. *AUC* Area under the curve, ^a^Under the non-parametric assumption, *Asym. sign.* Asymptotic significance (null hypothesis: true area = 0.5), *Asym. 95% CI* Asymptotic 95% confidence interval

**Fig. 3:**
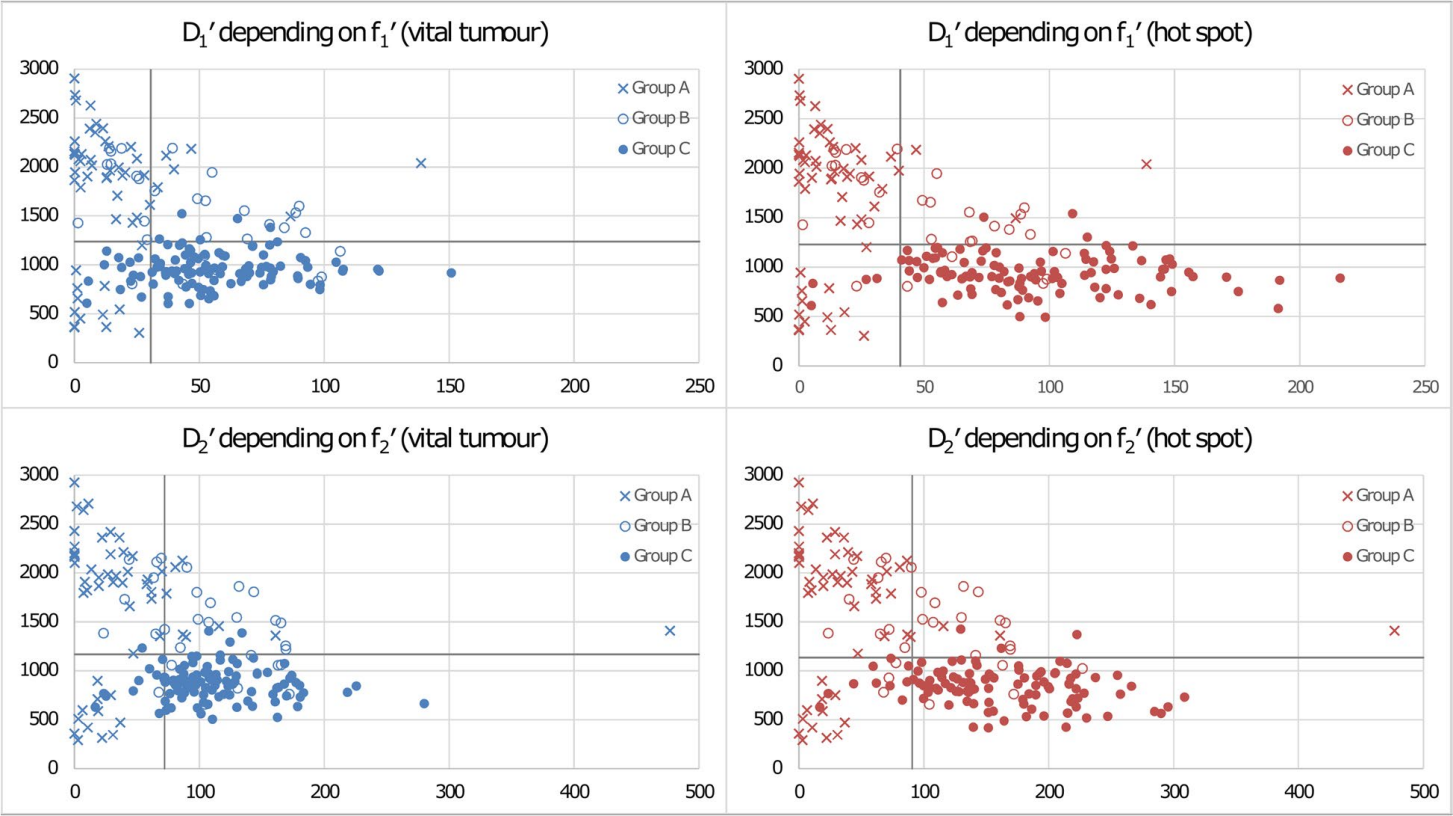
Overview to combined use of *D*_1_′ and *f*_1_′ as well as *D*_2_′ and *f*_2_′ parameters. On the ordinate axis *D*_1_′ respectively *D*_2_′ is given (in units of 10^-6^ mm^2^/s), on the abscissa axis *f*_1_′ respectively *f*_2_′ (in units of 10^-3^). Blue data points (left diagrams) belong to vital tumour regions of interest (VT-ROIs), red (right diagrams) to hot spot ROIs (HS-ROIs). For each parameter, the cutoff point (see Table 5) is also given (grey lines). It is obvious that differentiation between benign lesions (group A+B) and malignant lesions (group C) is comparable for *D*_1_′ and *D*_2_′ and for VT-ROIs and HS-ROIs. However, in the case of HS-ROIs, the differentiation improves clearly, if *f*_1_′ respectively *f*_2_′ is used together with *D*_1_′ respectively *D*_2_′ (lesions in the quadrant bottom right were assigned as malignant, all other lesions as benign), especially for combination *D*_1_′+*f*_1_′ with 93.7% correctly identified cases instead of 88.0% (see Table 5)

For VT-ROIs, the largest AUC values were reached for ADC, D_1_′, and D_2_′ (0.817, 0.819, and 0.826, respectively) (Tables 5 and 6). The diagnostic accuracy (Table 5) of the combinations *D*_1_′+*f*_1_′ (87.4%) and *D*_2_′+*f*_2_′ (89.0%) as described in the ‘Statistical analysis’ section (Fig. 3) were similar (*p* ≥ 0.414) to that of ADC (87.4%), *D*_1_′ (86.9%) and *D*_2_′ (86.9%).

**Table 6:** Results (*p* values) of comparisons between areas under the curve values presented in Table 5 for group A+B *versus* group C

Parameter	ADC	*D*_1_′	*D*_2_′	*f*_1_′	*f*_2_′	D*′
Vital tumour ROIs						
ADC		0.631	0.145	0.696	0.622	< 0.001
*D*_1_′			0.086	0.672	0.592	< 0.001
*D*_2_′				0.568	0.495	< 0.001
*f*_1_′					0.804	0.001
*f*_2_′						0.024
D*′						
Hot spot ROIs						
ADC		0.015	0.003	0.051	0.155	0.030
*D*_1_′			0.020	0.070	0.255	0.007
*D*_2_′				0.108	0.328	0.006
*f*_1_′					0.119	*<* 0.001
*f*_2_′						0.003
Hot spot ROIs *versus* vital tumour ROIs						
ADC	0.045					
*D*_1_′		0.022				
*D*_2_′			0.008			
*f*_1_′				*<* 0.001		
*f*_2_′					*<* 0.001	
D*′						*<* 0.001

*ADC* Apparent diffusion coefficient

**Table 7:** Results of the receiver operating characteristic analysis for the differentiation between benign and malignant lesions group B *versus* group C

Test variable	Numbers	AUC	Standard error^a^	Asym. sign.	Asym. 95% CI		Cutoff point	Sensitivity	Specificity	Accuracy
					Lower bound	Upper bound				
Vital tumour ROIs										
ADC	30 *versus* 105	0.868	0.048	< 0.001	0.772	0.964	1,279.8	0.924	0.800	0.896
*D*_1_′	30 *versus* 105	0.859	0.051	< 0.001	0.759	0.960	1,238.7	0.952	0.767	0.911
*D*_2_′	30 *versus* 105	0.870	0.047	< 0.001	0.775	0.964	1,055.2	0.867	0.833	0.859
*f*_1_′ #	30 *versus* 105	0.562	0.067	0.355	0.429	0.694				
*f*_2_′ #	30 *versus* 105	0.546	0.066	0.491	0.414	0.677				
D*′ #	30 *versus* 105	0.528	0.067	0.676	0.395	0.661				
*D*_1_′ + *f*_1_′	30 *versus* 105									
*D*_2_′ + *f*_2_′	30 *versus* 105									
Hot spot ROIs										
ADC	30 *versus* 105	0.858	0.051	< 0.001	0.758	0.959	1,327.2	0.952	0.767	0.911
*D*_1_′	30 *versus* 105	0.870	0.047	< 0.001	0.777	0.964	1,229.2	0.971	0.767	0.926
*D*_2_′	30 *versus* 105	0.883	0.044	< 0.001	0.797	0.970	1,056.6	0.905	0.800	0.881
*f*_1_′ #	30 *versus* 105	0.795	0.045	< 0.001	0.706	0.884	69.0	0.724	0.733	0.726
*f*_2_′ #	30 *versus* 105	0.751	0.049	< 0.001	0.653	0.849	109.3	0.819	0.600	0.770
D*′ #	30 *versus* 105	0.633	0.061	0.027	0.513	0.753	16,774.8	0.771	0.500	0.711
*D*_1_′ + *f*_1_′	30 *versus* 105						1,229.2/69.0	0.695	0.900	0.741
*D*_2_′ + *f*_2_′	30 *versus* 105						1,056.6/109.3	0.743	0.900	0.778

See Table 5 for abbreviations

For HS-ROIs, comparable AUC values were found for ADC, *D*_1_′, *D*_2_′, *f*_1_′ and *f*_2_′ (Tables 5 and 6). The obtained accuracy of *D*_1_′+*f*_1_′ (93.7%) was significantly higher than that of ADC (86.9%, *p* = 0.003), *D*_1_′ (88.0%, *p* = 0.007), *D*_2_′ (88.0%, *p* = 0.007), *f*_1_′ (87.4%, *p* = 0.004), and *f*_2_′ (84.3%, *p* < 0.001) and slightly but not significantly higher than that of *D*_2_′+*f*_2_′ (90.6%, *p* = 0.083).

Compared to the AUC values of VT-ROIs, the AUC values of HS-ROIs were clearly larger for *f*_1_′, *f*_2_′, and D*′ (*p* < 0.001), similar for ADC (*p* = 0.045) and slightly larger for *D*_1_′ (*p* = 0.022) and *D*_2_′ (*p* = 0.008) (Table 6). The accuracy of *D*_1_′+*f*_1_′ was significantly higher for HS-ROIs than for VT-ROIs (93.7% instead of 87.4%, *p* < 0.001), but not for *D*_2_′+*f*_2_′ (90.6% instead of 89.0%, *p* = 0.366).

Thus, the best discriminability was reached for *D*_1_′+*f*_1_′ using HS-ROIs with a sensitivity of 94.3% (95% CI 89.8−98.7%), a specificity of 93.0% (95% CI 87.6−98.4%), and an accuracy of 93.7% (95% CI 90.3−97.2%). Of 105 malignant lesions, 6 were falsely classified as benign due to high *D*_1_′ values (2 invasive lobular carcinomas with large diffuse propagation, 1 high-grade ductal carcinoma *in situ* [DCIS]) or low *f*_1_′ values (1 small invasive ductal carcinoma, 1 invasive carcinoma with accompanying inflammatory reaction and 1 lymph node metastasis). Of 86 benign lesions, 6, all of the group B, were falsely classified as malignant due to low *D*_1_′ in combination with high *f*_1_′ (3 intramammary lymph nodes, 1 sclerosing adenosis, 1 flat epithelial atypia and 1 syringomatous adenoma).

### IVIM as an add-on to DCE-MRI (group B *versus* group C)

The values of ADC, *D*_1_′, and *D*_2_′ were significantly smaller for group C compared to B, for VT- and for HS-ROIs, but the values of *f*_1_′, *f*_2_′, and D*′ were only for HS-ROIs significantly larger for group C compared to group A+B (Table 7).

For VT-ROIs, the AUC values of ADC, *D*_1_′, and *D*_2_′ (0.868, 0.859, and 0.870, respectively) were not significantly different (*p* ≥ 0.324) and the diagnostic accuracies (89.6%, 91.1%, and 85.9%, respectively) were similar (*p* = 0.317 for ADC *versus* *D*_1_′, *p* = 0.059 for ADC *versus* *D*_2_′, *p* = 0.035 for *D*_1_′ *versus* *D*_2_′) (Tables 7 and 8). There was a lack of significant differences between malignant and benign lesions in the perfusion parameters (*p* ≥ 0.355). Thus, the combinations *D*_1_′+*f*_1_′ and *D*_2_′+*f*_2_′ were not analysed.

**Table 8:** Results (*p* values) of comparisons between areas under the curve values presented in Table 7 for group B *versus* group C

Parameter	ADC	*D*_1_′	*D*_2_′	*f*_1_′	*f*_2_′	D*′
Vital tumour ROIs						
ADC		0.324	0.890			
*D*_1_′			0.328			
*D*_2_′						
*f*_1_′						
*f*_2_′						
D*′						
Hot spot ROIs						
ADC		0.191	0.075	0.323	0.138	0.002
*D*_1_′			0.198	0.192	0.081	0.002
*D*_2_′				0.119	0.035	< 0.001
*f*_1_′					0.288	0.013
*f*_2_′						0.188
D*′						
HS-ROI *versus* VT-ROI						
ADC	0.045					
*D*_1_′		0.022				
*D*_2_′			0.008			
*f*_1_′				*<* 0.001		
*f*_2_′					*<* 0.001	
D*′						*<* 0.001

*ADC* Apparent diffusion coefficient

For HS-ROIs, the largest AUC values were found for ADC, *D*_1_′, and *D*_2_′ (0.858, 0.870, and 0.883, respectively), which were not significantly different (*p* ≥ 0.075) (Tables 7 and 8). The accuracy (Table 7) of the combinations *D*_1_′+*f*_1_′ (74.1%) and *D*_2_′+*f*_2_′ (77.8%) were similar (*p* ≥ 0.297) and significantly lower than that of ADC (91.1%, *p* < 0.001 for both), *D*_1_′ (92.6%, *p* < 0.001 for both) and *D*_2_′ (88.1%, *p* = 0.001 and *p* = 0.002, respectively).

Compared to the AUC values of VT-ROIs, the AUC values of HS-ROIs were clearly larger for *f*_1_′, *f*_2_′, and D*′ (*p* < 0.001), slightly larger for *D*_1_′ and *D*_2_′ (*p* = 0.022 and *p* = 0.008) and similar for ADC (*p* = 0.045) (Table 8).

Thus, best discrimination was reached for single parameter *D*_1_′ using HS-ROIs with a sensitivity of 97.1% (95% CI 94.0−1.00%), a specificity of 76.7% (95% CI 61.5−91.8%) and an accuracy of 92.6% (95% CI 88.2−97.0%). Of 105 malignant lesions, 3 were falsely classified as benign due to high *D*_1_′ values (2 invasive lobular carcinoma with large diffuse propagation, 1 high-grade DCIS). Of 30 benign lesions/tissue, 7 were falsely classified as malignant due to low *D*_1_′ values (4 intramammary lymph nodes, 1 sclerosing adenosis, 1 flat epithelial atypia, 1 syringomatous adenoma).

## Discussion

In the present study, a detection rate (hyperintensity on DWI with *b* = 800 s/mm^2^) of 100% was reached for malignant lesions. In other studies, comparable detection rates to abbreviated DCE-MRI were also found [46–48], except for some tumours with non-mass enhancement, microcalcifications and small size [48–50]. Using simplified IVIM, the following results were reached for differentiation between benign and malignant breast lesions: (1) when including all conspicuous lesions on DWI with *b* = 800 s/mm^2^ (stand-alone tool), the best discriminability was reached for the combination *D*_1_′+*f*_1_′ using HS-ROIs (accuracy 93.7%), which was significantly higher than that of ADC (86.9%) and *D*_1_′ (88.0%) or *f*_1_′ (87.4%) alone; (2) when including only lesions with suspicious contrast-enhancement (add-on to DCE-MRI), the best diagnostic accuracy was reached for single parameter *D*_1_′ using HS-ROIs (92.6%), which were slightly but not significantly better than that of ADC (91.1%) and *D*_2_′ (88.1%). By adding *f*_1_′ to *D*_1_′, no improvement was reached.

The finding of lower *D* and higher *f* values in malignant lesions compared to benign lesions was also found by other authors [13–24]. It indicates higher cell density with reduced extracellular space and increased relative contribution of microvascular blood flow. By analysing perfusion hot spots, it was found that D* is only locally increased in malignant lesions. In other studies, inconsistent results were found for D* with lower [13, 15, 17, 19, 23] or higher [5, 14] values in malignancy, or hardly any difference [16, 18, 21, 51, 52]. On the other side, perfusion heterogeneity of breast cancers is well known [53]. In malignant lesions, 27% of the voxels (on average) showed no perfusion at all. In other studies, even more than 50% of the voxels showed no perfusion [31, 54]. Thus, a voxel-wise parameter calculation is important for analysing perfusion, even if a ROI-averaged signal analysis was preferred in some studies to facilitate bi-exponential fitting due to higher signal-to-noise ratio [22, 24, 55]. Angiogenesis is an important prognostic indicator of tumour growth, metastatic potential and response to adjuvant therapies [56].

In the present study, a perfusion hot spot analysis was performed, which has not published before to our knowledge. Some diffusion hot spot analyses showed better diagnostic performance in areas with most restricted diffusion compared to large ROI analysis [57–60] and also for minimum ADC or a low percentile compared to mean ADC [61]. In the present work, for diffusion parameters only weak differences were found between HS-ROIs and VT-ROIs, because both ROIs comprised only areas with hyperintensity on DWI with *b* = 800 s/mm^2^. Perfusion hot spots in areas of minimum diffusion are potentially the most active parts (proliferating cellularity and abundant angiogenic neovascularity), where biopsy should be made [21] (Fig. 1). In some lesions, however, the perfusion hot spot was not in an area of minimum diffusion, in agreement to previously published data [6] (Fig. 13).

The evaluation of IVIM-DWI as a stand-alone tool yielded a good diagnostic accuracy being better than that of ADC. In contrast to other studies [13, 14, 16, 19, 22, 23, 62], the benign group contained also complicated cysts, haematomas and intramammary lymph nodes. Such lesions often have low *D* values [63, 64] like malignant lesions, leading to false-positive assignments. Despite the inclusion of such lesions, the accuracy of single parameter *D*_1_′ (86.9–88.0%, cutoff 1.23–1.24×10^-3^ mm^2^/s) was in the range of other studies (75–91.3%, cutoff 1.01–1.21×10^-3^ mm^2^/s) [14, 16, 17]. In one study, a higher accuracy (96.8%) was reached [19], but the benign lesion group contained only fibroadenomas and papillomas. For HS-ROIs, the accuracy of ADC tended to be lower than of *D*_1_′, as found in other studies [15, 18, 19, 22].

We should note that, in contrast to *D*, the ADC is also influenced by perfusion, which enlarges the reduced values of malignant lesions in proportion to *f* and brings the values of malignant lesions closer to that of benign lesions. For *f*_1_′, for HS-ROIs better accuracy (87.4%, cutoff 0.041) was reached than for VT-ROIs (80.1%, cutoff 0.031) and for *f* in other studies (62.1−76.2%, cutoff 0.050−0.079) [14, 16, 17]. For the combined *D*_1_′+*f*_1_′ analysis, in case of HS-ROIs, higher diagnostic discriminability was obtained than for *D*_1_′ alone (93.7 *versus* 88.0%) due to improved specificity (93.0 *versus* 76.7%).

With *D*_1_′+*f*_1_′, liquid-filled lesions/compartments can be differentiated from malignant lesions by their uniformly low perfusion fraction. Only some malignant lesions were assigned as false negatives due to high *D*_1_′ or low *f*_1_′ as described above. In other studies, non-mass lesions [65], invasive ductal carcinoma, invasive lobular carcinoma, DCIS and mucinous carcinoma [22] were falsely assigned as benign. Some benign lesions were assigned as false positives due to low *D*_1_′ AND high *f*_1_′. In other studies, lobular carcinoma *in situ*, adenosis lesions and intraductal papilloma were false positives [17, 22]. Improved accuracy was also reached in another three *b* value studies for a diffusion- and perfusion-weighted parameter, *i.e.*, RED = ADC_perf_/*D*, with *D* calculated as ADC for *b* values of 200 and 700 s/mm^2^ and ADC_perf_ calculated as ADC for *b* values of 0 and 200 s/mm^2^ minus *D*, compared to *D* (90.0 *versus* 86.7%) [62]. However, the reached diagnostic performance (88.2% sensitivity, 92.3% specificity, and 90% accuracy) was lower than that of the present study (94.3%, 93.0%, and 93.7%, respectively), although not even cysts, haematomas and intramammary lymph nodes were included. The same applies of the improvements in DCE-MRI by analysing the dynamic parameter time to enhancement derived from ultrafast breast MRI instead of conventional curve type evaluation (94%, 79%, and 87% *versus* 91%, 53%, and 72%, respectively) [66]. This suggests that IVIM-DWI is an appealing alternative to DCE-MRI for breast cancer screening at least in patients in whom contrast agents are contraindicated, in regularly monitored patients to avoid repetitive gadolinium applications, and in patients whose breasts show marked background parenchymal enhancement on DCE-MRI [67].

The evaluation of IVIM-DWI as add-on to DCE-MRI showed that the diagnostic accuracy could not be improved by perfusion analysis. To date, DWI with ADC calculation is the most widely explored adjunct to reduce false positives on conventional DCE-MRI [6, 8]. In the present study, it was shown that for lesions with suspicious contrast-enhancement the diagnostic performance tended to be higher for *D*_1_′ than for ADC due to higher sensitivity (97.1% sensitivity, 76.7% specificity, and 92.6% accuracy *versus* 95.2%, 76.7%, and 91.1% in the case of HS-ROIs, respectively). For *D*_1_′, a lower number of DCIS and mucinous invasive ductal carcinomas appeared as false negative. With *D*_1_′ as add-on, 76.7% of unnecessary biopsies in patients with benign lesions could be prevented with minimal loss of sensitivity compared to DCE-MRI alone. Other studies with IVIM-DWI as add-on to DCE-MRI are rare [5, 7] and showed worse results: 99.1% sensitivity, 56.5% specificity, and 77.8% accuracy [7]; 88.9%, 85.1%, and 87.5%, respectively [5].

To our knowledge, simplified IVIM in application to breast lesions has been only evaluated in one initial study [3]. In that study, better or similar diagnostic performance was found with simplified IVIM with explicit formulas for *D* and *f* determination than with a 12-*b*-value fitting approach. However, a patient cohort of only 26 patients was investigated and only one *3*-*b*-value approach (*b* = 0, 200, and 800 s/mm^2^) was used. In the present study, a larger patient cohort of 126 patients was evaluated. In addition, two different 3-*b*-value combinations (*b* =0, 50, 800 s/mm^2^ and 0, 250, 800 s/mm^2^) were compared and the added value a 4-*b*-values approach was evaluated. The 4-*b*-value approach yielded no added value. For *b* = 0, 50, 800 s/mm^2^, a higher diagnostic accuracy was reached than for *b* = 0, 250, 800 s/mm^2^. Moreover, the evaluation of simplified IVIM in the present study yielded that this approach is particularly suitable for clinical application due to its low acquisition time of less than 3 min and the simplified analysis by using explicit formulas without any fitting procedure. The analysis of only two parameters (*D*_1_′ and *f*_1_′) is sufficient. In order to further simplify and speed up the assessment procedure, the evaluation of so-called two-colour index maps, already successfully used for liver lesions [68], is planned.

A limitation of the present study is that reproducibility of ROI placement has not been investigated. There is also a lack of validation of the results with the help of an independent patient group, which is planned for a next study. In the present study, a maximum *b* value of 800 s/mm^2^ was used, as recommended by the international breast DWI working group [2]. Higher maximum *b* values may lead to higher non-Gaussian influences [69] and noise-biased effects [21, 29] while lower *b* values are more influenced by perfusion [59]. Typically, thresholds of 150–400 s/mm^2^ were used [16, 17, 20–22, 24, 30, 31, 52]. *f* values calculated from *b* = 50 s/mm^2^ might be also influenced by D*, but may serve successful as an empirical marker for the perfusion effects. *f* may vary with used echo time and repetition time due to its dependence on T1 and T2 relaxation times [70]. However, for diagnostic differentiation, high precision of parameter measurement is more important than high accuracy [55]. As in some other studies [46, 48], short-time inversion recovery instead of spectral-selective fat-suppression was used due to its superiority in avoiding partial volume effects and signal overlay in relation to large water-fat-shift in single-shot DWI [71], fat suppression homogeneity [72, 73], lesion detectability [74] and measurement reproducibility of ADC [71], if used before gadolinium contrast agent application [73]. In contrast to fatty breasts, dense breasts did not affect lesion detectability and ADC values [75]. It was found that for DWI, expert-level readers are necessary for reaching good results [76], because the detection and avoidance of areas affected by artefacts is important for parameter analysis and requires some experience. In general, DWI is not suited for patients with implants and in case of small lesions, which is a potential source of bias. Another bias is given by patient selection including many high-risk patients, because a suspicious lesion was previously found. Thus, the application as screening tool needs further investigation.

In conclusion, IVIM analysis of lesions ≥ 8 mm yielded a higher diagnostic accuracy than ADC in terms of malignant *versus* benign differentiation of breast lesions. Perfusion analysis appeared of special relevance, if DWI is used as stand-alone tool.

## Data Availability

The datasets used and/or analysed during the current study are available from the corresponding author on reasonable request.

## Abbreviations

ADC: Apparent diffusion coefficient
AUC: Area under the curve
CI: Confidence interval
DCE: Dynamic contrast-enhanced magnetic resonance imaging
DCIS: Ductal carcinoma *in situ*
DWI: Diffusion-weighted imaging
HS-ROI: ‘Hot spot’ region of interest
IQR: Interquartile range
IVIM: Intravoxel incoherent motion
MRI: Magnetic resonance imaging
ROC: Receiver operating characteristic
ROI: Region of interest
VT-ROI: ‘Vital tumour’ region of interest
